# Synthetic Lethal Combinations of DNA Repair Inhibitors and Genotoxic Agents to Target High‐Risk Diffuse Large B Cell Lymphoma

**DOI:** 10.1002/hon.70131

**Published:** 2025-08-23

**Authors:** Sara Ovejero, Julie Devin, Laura Alibert, Camille Soun, Yea‐Lih Lin, Laure Dutrieux, Matthieu Abouladze, Elvira Garcia de Paco, Ouissem Karmous Gadacha, Angelos Constantinou, Guillaume Cartron, Charles Herbaux, Olivier Elemento, Philippe Pasero, Sandrine Roulland, Jérôme Moreaux, Caroline Bret

**Affiliations:** ^1^ Department of Biological Hematology CHU Montpellier Montpellier France; ^2^ IGH Univ Montpellier, CNRS, CHU Montpellier Montpellier France; ^3^ Aix Marseille University CNRS INSERM Centre d'Immunologie de Marseille‐Luminy Marseille France; ^4^ Englander Institute for Precisiom Medicine Institute for Computational Biomedicine Weill Cornell Medical College New York New York USA; ^5^ Department of Clinical Hematology CHU Montpellier Montpellier France; ^6^ University of Montpellier University of Medicine Montpellier France; ^7^ Institut Universitaire de France (IUF) Paris France

**Keywords:** biomarker, CRISPR‐Cas9, DLBCL, DNA damage, DNA repair, genotoxic stress, inhibitors

## Abstract

Diffuse large B‐cell lymphoma (DLBCL) is the most common hematological malignancy. More than half of DLBCL patients achieve long‐term remission after treatment, but a third relapse after conventional Rituximab (R)‐based chemotherapy regimens, such as CHOP (cyclophosphamide, doxorubicin, vincristine and prednisone). Cancer cells are exposed to chronic replication stress, which impedes the duplication of their genome. Functional DNA repair pathways are therefore important for the survival of cancer cells. This dependence can be exploited therapeutically to hamper repair of the intrinsic DNA damage occurring during replication or to exacerbate DNA damage induced by chemotherapy. Using CRISPR‐Cas9 screening, we identified *CHEK1, WEE1, ATR* and *RAD51* DNA repair factors as essential genes in DLBCL cells. According to these results, we investigated the effect of small molecules targeting DNA replication stress and DNA repair mechanisms, alone or in combination with the R‐CHOP genotoxic agents, cyclophosphamide and doxorubicin. Applying a standard threshold of 2 SDs below the IC50 of the genotoxic agent alone, a total of 3 synthetic lethal combinations have been identified including cyclophosphamide with CHK1/2 inhibitor, cyclophosphamide and ATR inhibitor and doxorubicin with DNAPK inhibitor. Co‐treatment with these molecules led to cell death, DNA damage induction and cell cycle arrest in DLBCL cells more efficiently than genotoxic agents alone. These data have been validated using primary DLBCL cells from patients. Our results open new perspectives for therapeutic approaches exploiting the synthetic lethality of genotoxic agents with DNA repair inhibitors to improve the therapeutic outcome of patients with DLBCL.

## 1 Background

Diffuse large B‐cell lymphoma (DLBCL) is the most common type of non‐Hodgkin lymphoma (NHL) worlwide. DLBCL is primarily treated with R‐CHOP, a regimen of rituximab, cyclophosphamide, doxorubicin, vincristine and prednisone [1]. However, a substantial fraction of patients with relapsed/refractory disease continue to die from this disease of the associated complications. Based on the cell of origin, two main gene‐expression based‐subgroups have been identified including the germinal center B‐cell group (GCB) and the activated B‐cell group (ABC). GCB and ABC account for 50% and 30% of DLBCL cases, respectively, and the remaining 20% of patients present rare or unclassifiable types of large B‐cell lymphomas [2, 3]. GCB and ABC are heterogeneous diseases, and are associated with different prognosis and responses to treatment, with ABC being more aggressive and having a poorer prognosis [4]. More recently, large‐scale genomic studies have identified genetic subtypes of DLBCL based on shared genomic abnormalities, a classification i.e. not necessarily overlapping with the COO subgroups [5, 6, 7]. Finally, a transcriptomic analysis of the microenvironment based on deconvolution of large DLBCL cohorts revealed four distinct microenvironment compositions associated with distinct clinical behavior and biological subtypes of DLBCL [8].

Currently, R‐CHOP achieves cure in approximately 60% of DLBCL patients [1]. Unfortunately, 40% of DLBCL patients have primary refractory disease or relapse shortly after remission (i.e., therapy‐resistant) and continue to die from this disease. The development of novel targeted approaches to treat these cases is a pressing need. Cyclophosphamide is an intercalating agent that generates interstrand crosslinks (ICLs) and DNA damage [9]; doxorubicin is an anthracycline that stabilizes the Topoisomerase II on DNA stopping replication and causing DNA damage [10]; vincristine is a spindle poison that impairs cell division [11]; and prednisone is a glucocorticoid. Thus, genotoxic stress induced by cyclophosphamide and doxorubicin is an important part of the R‐CHOP regimen. In salvage therapies, other agents inducing DNA damages could be used including the nucleoside analog gemcitabine [12] and etodposide [13, 14]. Etoposide inhibits topoisomerase II activity by forming a ternary complex with DNA inducing DNA single‐ and double‐strand breaks [15]. We have previously developed GEP‐based DNA repair signatures that allow the identification of 20% of high‐risk DLBCL patients and highlight the potential role of DNA damage response (DDR) in DLBCL drug resistance [16]. A list set of 176 genes involved in six major DNA repair pathways including base excision repair (BER), NER, mismatch repair (MMR), homologous recombination repair (HRR), non‐homologous end joining (NHEJ) and FANC pathways was analyzed using GEP data from newly diagnosed DLBCL patients treated by R‐CHOP [16]. We showed that 126 out of the 176 genes have a prognostic value with 92 genes associated with a poor outcome. For each pathway, a gene expression profile (GEP)‐based risk score was created. High FANC, NER, HRR, BER, NHEJ and MMR scores were significantly associated with poor prognosis in the two independent cohorts of patients with DLBCL underlining a potential role of DNA repair pathways in the pathogenesis and drug resistance of DLBC [16]. Genetic instability is a hallmark of cancer cells that appears at an early stage and increases during the course of the disease. One of the main causes of genetic instability is replication stress and its associated DNA damage. In fact, perturbations in DNA replication can lead to under or over‐replication, accumulation of point mutations, and chromosome instability among others, which challenge cell survival and are at the origin of malignant transformation and progression [17, 18, 19]. Importantly, replication stress has also been proposed as an Achilles' heel of cancer cells, since they are often addicted to DNA damage repair (DDR) pathways, which opens the door to therapeutic strategies that exploit the concept of synthetic lethality. Synthetic lethality relies on the fact that DNA lesions can be processed by different repair pathways depending on their nature. When all the pathways required to repair a particular type of lesion are not functional, the result is a catastrophic accumulation of DNA damage, either intrinsic or caused by chemotherapy, that ultimately leads to cell death. Hence, synthetic lethality provides a strong rationale for the combination of genotoxic agents with inhibitors of the main DDR pathways to treat cancer [20, 21, 22].

Here, we used previously published genome wide pooled CRISPR‐Cas9 screens data performed on 4 ABC and 4 GCB‐DLBCL cell lines [23] and assessed the enrichment/depletion score of 126 genes related to DNA repair pathways [16]. We identified the DNA repair factors *CHEK1*, *WEE1*, *ATR*, and *RAD51* as essential genes in DLBCL cell lines. DNA damage induces the activation of ATR (ataxia‐telangiectasia‐mutated‐and‐Rad3‐related kinase) and its downstream effector kinase Chk1 to arrest cell cycle progression and prevent the entry into mitosis with incompletely replicated or damaged DNA. Thus, ATR, WEE1 and Chk1 inhibitors have been developed and their therapeutic potential in cancer is being assessed [24, 25, 26, 27, 28]. Wee1 kinase is a cell cycle regulator that controls the timely entry into mitosis by inhibiting the Cdk1‐Cyclin B1 complex [29]. In addition, Wee1 is activated by Chk1 in response to DNA damage to allow DNA repair before the G2/M transition. It has been reported that treatment with Wee1 inhibitor leads to unscheduled entry into mitosis without proper DNA repair that results in mitotic catastrophe and cell death [30]. Rad51 is a recombinase involved in the repair of DSB (DNA double strand breaks) by homologous recombination (HR) that is commonly dysregulated in cancer [31]. Several Rad51 inhibitors have been evaluated or are under development to target cancer cells with promising results [32]. Importantly, previous work reported that the chemical inhibition of Chk1 and Wee1 may be a good therapeutic approach in DLBCL. On the one hand, the inhibition of Wee1 by AZD‐1775 has been shown to synergize with the CHOP chemotherapy and with radiation therapy to kill DLBCL cells [33]. On the other hand, the combination of Chk1 and Wee1 inhibitors strongly reduced Myc protein levels and led to cell death in lymphoma cell lines [34, 35]. Of note, the combination of both inhibitors also led to increased γH2AX levels in seven DLBCL cell lines, indicative of DNA damage induction [35]. Similarly, the response of non‐GCB DLBCL cells to ATR and Wee1 inhibitors was shown to be linked to replication stress [36]. Furthermore, ATRi elimusertib demonstrated ati‐lymphoma activity in vitro and in vivo and synergized with PI3K inhibitor [37, 38]. Taken together, these data point at a great interest of DNA repair factors as chemotherapeutic targets in DLBCL.

Synthetic lethality occurs when mutations in two genes simultaneously result in cell death, while individual mutations in either gene alone allow cells to survive [39]. Years later, this concept was adapted for cancer research, ultimately culminating in the development and approval of novel therapeutic approaches [40]. Drug‐drug synthetic lethality was designed to overcome inherent drug resistance, which frequently occurs when drug treatment triggers feedback mechanisms that activate the targeted pathway or compensatory parallel pathways [39, 40]. Based on these results, we investigated the effect of small molecules targeting DNA replication stress and DNA repair mechanisms, alone or in combination with the R‐CHOP genotoxic agents, cyclophosphamide and doxorubicin. Moreover, we found three synthetic lethal combinations: (1) cyclophosphamide plus the Chk1/2 inhibitor PF‐477736, (2) cyclophosphamide and the ATR inhibitor AZD‐6738, and (3) doxorubicin with the DNA‐PK inhibitor NU‐7441. Co‐treatment with these molecules led to cell death in cell lines and primary DLBCL cells from patients by increasing DNA damage more efficiently than genotoxic agents alone. Our findings open new perspectives for therapeutic approaches exploiting the synthetic lethality of genotoxic agents with DNA repair inhibitors to improve the therapeutic outcome of patients with DLBCL.

## 2 Materials and Methods

### 2.1 Drugs and Inhibitors

The compounds used in this study are PJ‐34 (PARP1/2 inhibitor; 3255 Tocris Bioscience), NU‐7441 (DNAPK inhibitor; S2638 Selleckchem), KU‐55933 (ATM inhibitor; S1092 Selleckchem), PF‐477736 (CHK1/2 inhibitor; S2904 Selleckchem), AZD‐6738 (ATR inhibitor; S7693 Selleckchem), MK‐8776 (CHK1 inhibitor; S2735 Selleckchem), AZD‐1775 (Wee1 inhibitor; S1525 Selleckchem), MP‐470 (Rad51 inhibitor; S1244 Selleckchem), Gemcitabine (nucleic acid synthesis inhibitor; S1714 Selleckchem), Doxorubicin and Etoposide (topoisomerase II inhibitors; D1515 Sigma Aldrich and S1225 Selleckchem, respectively), Mafosfamide (nitrogen mustard, active form of 4‐OH‐Cyclophosphamide; SC‐211761 Santa Cruz Biotechnology.

### 2.2 Culture of DLBCL Cell Lines

The 16 DLBCL cell lines from the 2 subtypes: ABC‐DLBCL (NU‐DUL‐1, OCI‐LY3, RI‐1, U‐2932) and GCB‐DBCL (DB, DOHH‐2, HT, NU‐DHL‐1, OCI‐LY1, OCI‐LY7, OCI‐LY19, SU‐DHL‐4, SU‐DHL‐5, SU‐DHL‐6, SU‐DHL‐10, WSU‐DLCL‐2) were purchased from the DSMZ (Leibniz‐Institut DSMZ ‐ Deutsche Sammlung von Mikroorganismen und Zellkulturen GmbH, Germany). Cells were maintained in RPMI‐1640 (Gibco, Invitrogen), supplemented with 10% fetal bovine serum (PAA laboratory GmbH) for U2932, SUDHL‐4, HT, DOHH2, SUDHL‐10, RI‐1, and WSU‐DLCL‐2 cell lines, and 20% FBS OCI‐LY3, DB, SUDHL‐5, NU‐DHL‐1, NU‐DUL‐1 and SU‐DHL‐6, cell lines. OCI‐LY1, OCI‐LY7 was cultured in IMDM (Gibco, Invitrogen), supplemented with 20% fetal bovine serum and OCI‐LY19 was cultured in MEM alpha modified (Gibco, Invitrogen), supplemented with 20% fetal bovine serum. Cultures were maintained at 37°C in a humidified atmosphere with 5% CO_2_.

### 2.3 Culture and Treatment of Primary Cells and FACS

Lymph node samples were collected after patients' written informed consent in accordance with the Declaration of Helsinki and institutional research board approval from Montpellier University Hospital (HEMODIAG_2020 cohort (ID‐RCB: 2011‐A00924‐37, NCT02134574)). Frozen cells in 10% DMSO from lymph nodes of 5 patients with DLBCL banked in the HEMODIAG_2020 collection were thawed, washed and qualified by flow cytometry.

For drug sensitivity assays, primary cells were cultured for 96 h in Gibco Iscove's MDM (Glutamax) medium (#31980‐022) with 20% FBS with antibiotics‐antimycotics 1X (Gibco Penicillin‐streptomycin‐amphotericin B 100X, #15240‐096) at a density of 0.5 × 10^6 Cell/mL with 50 ng/mL of histidine‐tagged CD40 L (R&D System, 2706‐CL) and 5 µg/mL of anti‐histidine antibody (R&D System, MAB050) and pyruvate 1X (Gibco pyruvate 100X, # 1136‐039).

Total cells were counted with trypan blue and stained with the panel CD45 V500 (BD, #560777), Ig Kappa FITC (Dako, F0434), CD19 PE‐Cy7 (BD, #341113), Ig Lambda PE (Dako, R0437), CD3 APC‐H7 (BD, #641415), CD10 APC (BD, #332777) and CD20 V450 (BD, #655872) and analyzed by flow cytometry (Canto II cytometer, Becton Dickinson).

The percentage of tumor cells (CD19+, CD45+, CD20+, Igκ or Igλ, CD10+ (if GCB subset)), non‐tumor cells (CD45+, CD19‐), and T cells (CD45+, CD3+) in each sample was determined by flow cytometry before and after treatment.

### 2.4 Loss‐Of‐Function CRISPR‐Cas9 Genetic Screens

The genome‐wide CRISPR‐Cas9 loss‐of‐function screening data were collected from Phelan et al. [23] and include 4 GCB and 4 ABC DLBCL cell lines. A CRISPR Screen Score (CSS) for DNA repair genes was calculated as previously described in the same work. CSS is a normalized value of toxicity considering all 4 sgRNA for each gene present in the CRISPR library. The results were also validated using publicly available database from Dependency Map portal (Broad Institute, www.depmap.org) [41].

### 2.5 Determination of the IC50 of Drugs and Inhibitors by Cell Viability Assays

DLBCL‐derived cell lines were cultured for 4 days in 96‐well flat‐bottom microtiter plates in the presence or absence of the different inhibitors as decribed [42]. The number of viable cells in culture was determined using the CellTiter‐Glo Luminescent Cell Viability Assay (G7573 Promega) using a Centro LB 960 luminometer (Berthold Technologies). This test is based on quantitation of the intracellular ATP present, which signals the presence of metabolically active cells. Data are expressed as the mean percentage of six technical replicates, normalized to the untreated control.

### 2.6 Statistical Analysis

Statistical tests were performed with Graphpad Prism V7 (Graphpad Software) and R i386 V3.4.0 software (R Fondation). Inhibitory concentration 50 (IC50) was calculated with the four‐parameter logistic regression formulA, as previously described [42, 43].

Significant synergy and combination indexes (CI) were calculated using the Chou‐Talalay formula [44] as previously described [43, 45].

## 3 Results

### 3.1 Analysis of the Therapeutic Interest of Drugs Targeting the DDR and Genotoxic Agents in the Treatment of DLBCL

**FIGURE 1:**
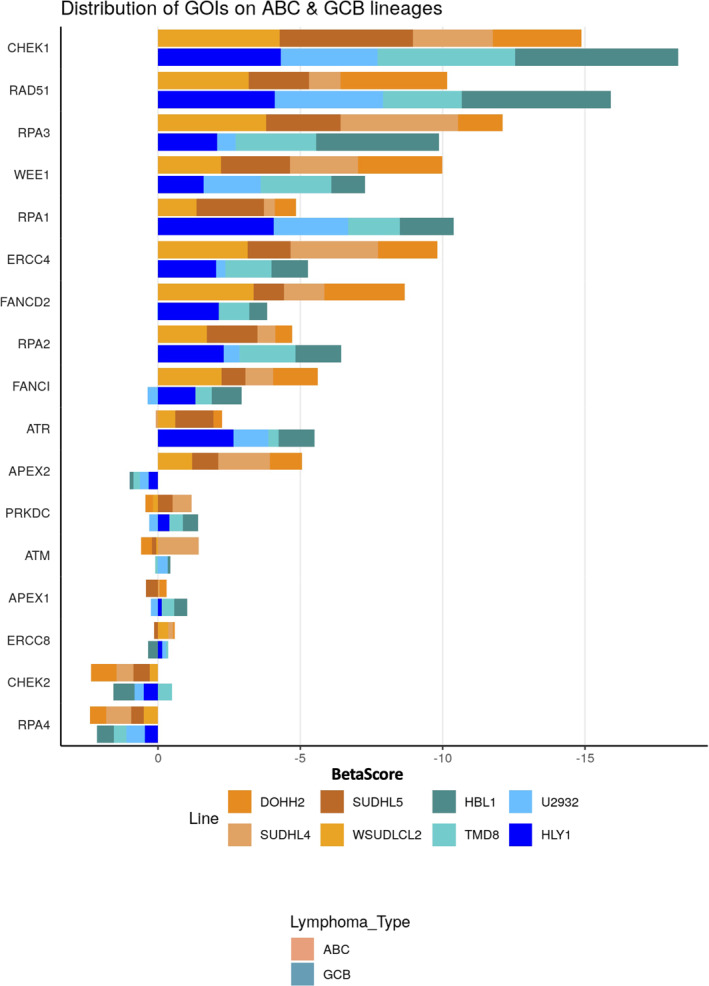
DDR genes are essential genes in DLBCL cells. Cumulative plot showing the dependency scores calculated using data from 8 cell lines [23]. A negative score indicates that gene is more likely to essential in DLBCL cell lines.

In an effort to better understand the role of the DNA damage repair (DDR) pathways in DLBCL [16] and their therapeutic interest, we mined previously published genome wide pooled CRISPR‐Cas9 screens data performed on 4 ABC and 4 GCB‐DLBCL cell lines [23] and assessed the enrichment/depletion score of 126 genes related to DNA repair pathways [16]. A negative score is associated with genes whose inactivation leads to cell death. We identified that sgRNAs targeting *ATR*, *CHEK1*, *WEE1*, ATR, RPA3 and *RAD51* were the most depleted indicating essentiality in DLBCL cell lines (Figure 1).We did not identified a specific dependency for ABC or GCB cell lines except for APEX2 that is only essential in ABC DLBCL cell lines. These results were confirmed using independent data from the DepMap portal (Figure S1).

**FIGURE 2:**
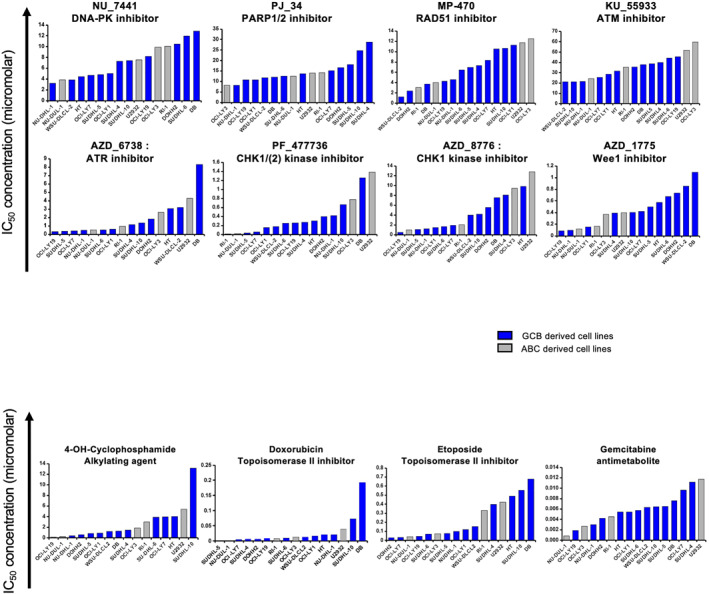
Characterization of IC_50_ of 10 drugs targeting DNA repair and 4 genotoxic agents in DLBCL cell lines. DLBCL cell lines were cultured for 96 h with culture medium (control) and increasing concentrations of the indicated drugs. The IC50 for each drug and each cell line was calculated with the four parameters logistic regression method. The IC50 represented here is the mean of at least three independent experiments.

Thus, we investigated the therapeutic interest of 8 DNA repair inhibitors targeting ATM (KU‐55933), ATR (AZD‐6738), Chk1/2 (PF‐477736), Chk1 (AZD‐8776), Wee1 (AZD‐1775), Rad51 (MP‐470), DNA‐PK (NU‐7441), and PARP (PJ34). As controls, the response to 4 conventional chemotherapy genotoxic agents (gemcitabine, 4‐OH‐Cyclophosphamide, etoposide, and doxorubicin) was assessed in 16 DLBCL cell lines (Figure 2 and Supporting Information S1). As expected, the 4 genotoxic agents presented a dose‐response in the order of nanomolar. Of them, the most genotoxic one was the nucleoside analog gemcitabine, which could be used as salvage therapy in relapsed/refractory DLBCL [12]. Inhibitors for DNA repair factors targeting Wee1 (AZD_1775), Chk1 (AZD_8776) and ATR (AZD_6738) showed a dose‐response ratio in the nanomolar order, while the rest of them presented a median IC50 in the micromolar range (Figure 2 and Supporting Information S1). In a previous work, we developed GEP‐based DNA repair signatures allowing the identification of high‐risk DLBCL patients and underlining the potential role of DDR in DLBCL drug resistance [16]. For each pathway, a gene expression profile (GEP)‐based risk score was created. High FANC, NER, HRR, BER, NHEJ and MMR scores associated with overexpression of genes involved in these pathways were significantly associated with poor prognosis in independent cohorts of patients with DLBCL. Of interest, we identified that high HRR and BER GEP‐based scores are associated with sensitivity to etoposide in the investigated cell lines (*r* = −0.58 and *r* = −0.70, respectively, *p* < 0.05). Furthermore a significant negative correlation between FANC score and the response to the ATR inhibitor AZD‐6738 (*r* = −0.58, *p* < 0.05) was also identified. These data indicate that high‐risk DLBCL patients identified with GEP‐based FANC and HRR/BER scores may benefit from treatment by ATR inhibitors and etoposide, respectively (Figure S2). However, high HRR score is associated with resistance to PARPi and high NHEJ score value coorelated with high IC50 of DNA‐PKi and ATMi (Figure S2).

In conclusion, our analyses show that deregulation of the DDR is a major mechanism for replicative stress adaptation and resistance to chemotherapy in DLBCL that could represent a therapeutic target.

### 3.2 Pharmacological Inhibition of ATR, Chk1 and Wee1 Induces Cell Death in DLBCL Cell Lines

**FIGURE 3:**
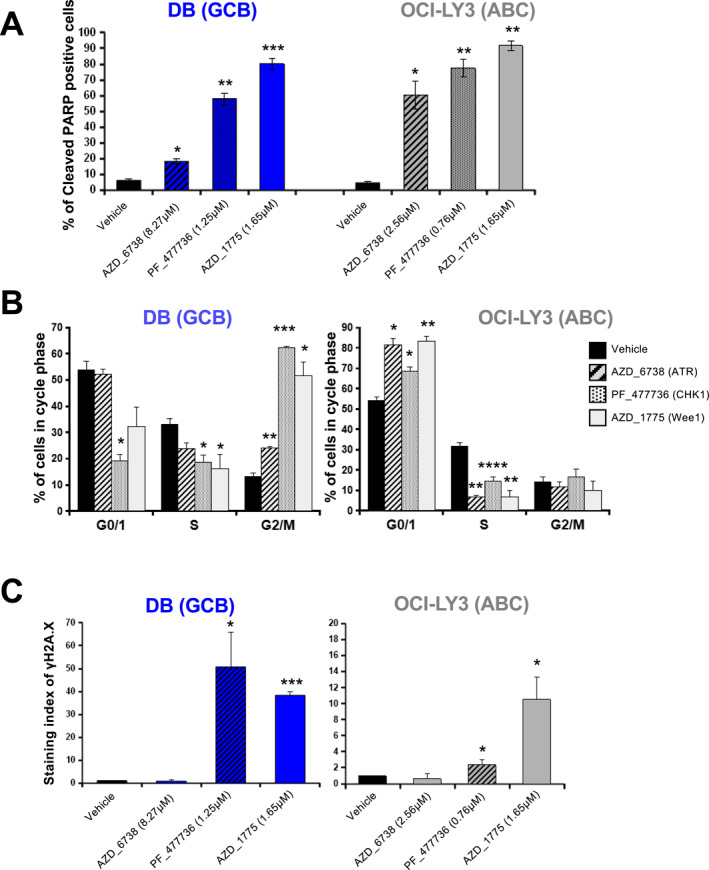
Effect of DDR inhibitors on apoptosis, cell cycle and DNA damage induction in DLBCL cells. (A, B) Cells were treated for 72 h. (A) Apoptotic cells (positive for cleaved‐PARP‐PE staining) were quantified by flow cytometry. (B) Cell cycle was analyzed using flow cytometry. S phase was monitored using BrdU incorporation and anti‐BrdU antibody. DNA content was stained with 4′,6‐diamidino‐2‐phenylindole (DAPI). Histograms represent the mean percentage and SEM of each cell cycle phase of three independent experiments. *p < 0.05, **p < 0.01 with paired Student t test. (C) Cells were incubated with AZD_6738, PF_477736 or AZD_1775 for 24H. All data in this figure represent the mean +/− SEM of three independent experiments. Statistical significance was tested using *t*‐test of pairs. *p < 0.05, **p < 0.01.

The CRISPR‐Cas9 screening data identified *ATR*, *CHEK1* and *WEE1* as essential genes in DLBCL cell lines (Figure 1 and Figure S1). Accordingly, the chemical inhibitors of ATR, Chk1 and Wee1 showed the most potent cytotoxicity in DLBCL cells correlating the CRISPR screen results (Figure 2). Thus, we aimed to further characterize the toxicity of AZD‐6738 (ATRi), PF‐477736 (Chk1i) and AZD‐1775 (Wee1i) using the cell lines OCI‐LY3 and DB, which belong to the ABC‐ and GCB‐DLBCL subtypes, respectively. PF‐477736 Chk1i was selected since it was reported to inhibit proliferation in lymphoma cell lines in vitro [46] and in MYC driven lymphoma model [47]. Treatment with the inhibitors of ATR, Chk1 and Wee1 induced a significant increase in PARP cleavage, indicative of apoptosis in the 2 lines (Figure 3A). This increase in cell death correlated with a decrease in proliferation marked by reduced S phase in both cell lines, and accumulation in G2/M phase for DB cells and in G0/G1 phase for OCI‐LY3 cells (Figure 3B). Interestingly, Chk1 and Wee1 inhibitors led to a significant increase of γH2AX positive cells, indicating DNA damage (Figure 3C). On the other hand, the genotoxic agents etoposide, doxorubicin and 4‐OH‐Cyclophosphamide, induced a marked increase in apoptosis and cell cycle arrest, also characterized by S‐phase arrest and G0/G1 or G2/M accumulation depending on the cell line (Figure S3AB). Doxorubicin caused DNA damage in both cell lines as determined by γH2AX staining, while 4‐OH‐Cyclophosphamide did so only in the DB cell line and etoposide did not cause significant DNA damage in either cell line (Figure S3C). Moreover, Chk1 and Wee1 inhibitors iduced caspase and PARP cleavage in DB together with phosphorylation together with DNA damage monitored by γH2AX induction in DB cell line (Figure S4). ATRi iduced Chk2 and p53 phosphorylation in DB cell line (Figure S4). In OCI‐LY3, ATRi, Chk1i and Wee1i induced caspase 8 cleavage and γH2AX induction (Figure S4). These results suggest that chemical inhibition of the DNA repair factors ATR, Chk1 and Wee1 or treatment with the genotoxic agents doxorubicin and 4‐OH‐Cyclophosphamide induce replication stress, leading to DNA damage and cell cycle arrest in S‐phase, which in combination may result in significant synergistic effects.

### 3.3 ATR, Chk1 and DNA‐PK Inhibitors Synergize With Genotoxic Agents to Kill DLBCL Cells

**FIGURE 4:**
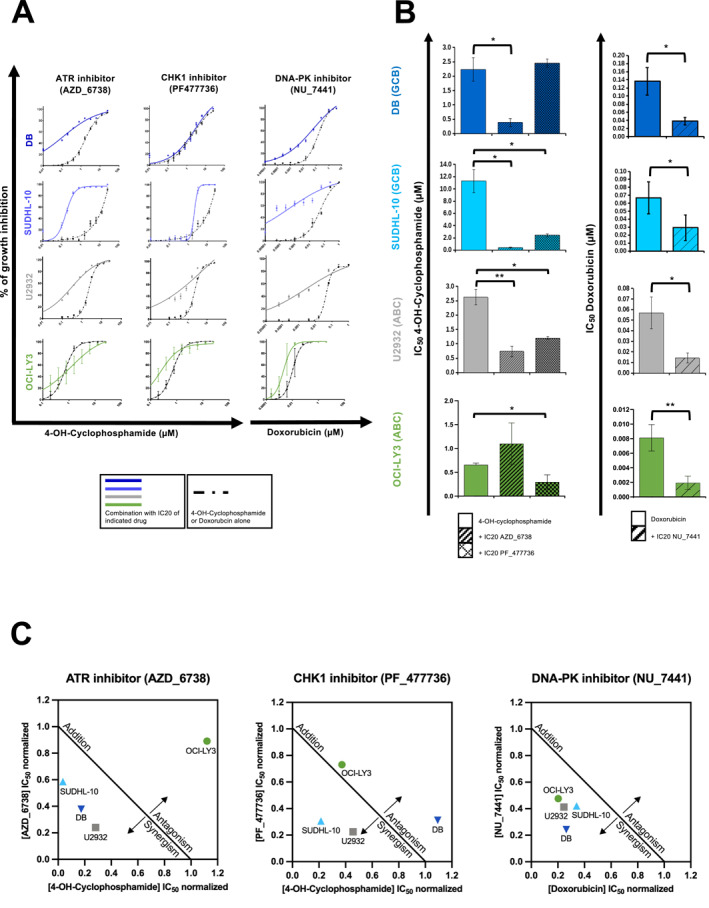
Synthetic lethal combination of DDR inhibitors with cyclophosphamide and doxorubicin in DLBCL cells. (A) DLBCL cell lines were treated with 4‐OH‐Cyclophosphamide in combination with IC20 of ATR inhibitor (AZD_6738) or IC20 of CHK1 inhibitor (PF_477736). The combination of Doxorubicin with the IC20 of DNA‐PK inhibitor (NU_7441) was also investigated in four DLBCL cell lines (DB, OCI‐LY3, U2932 and SUDHL10). IC50 were calculated after viability assessment by CellTiter‐Glo luminescent cell viability assay. Results are representative of three independent experiments. Significant synergy and combination index (CI) are calculated by the method of Chou Talalay. (B) Inhibitory concentration 50 of 4‐OH‐Cyclophosphamide in combination with IC20 of ATR inhibitor (AZD_6738) and CHK1 inhibitor (PF_477736). Inhibitory concentration 50 of doxorubicin combined with IC20 of DNA‐PK inhibitor (NU_7441). IC50 were calculated after viability assessment by CellTiter‐Glo luminescent cell viability assay. Results are representative of three independent experiments. (C) Significant synergy and combination index (CI) are calculated by the method of Chou Talalay. Normalized IC50 isobolograms showing the drug combination effect in the DLBCL cell lines. Statistical significance was tested using *t*‐test for pairs. *p < 0.05, **p < 0.01.

Since deregulation of DNA repair pathways plays a crucial role in drug resistance in many cancers [48], including DLBCL, we sought to identify new synthetic lethal combinations between inhibitors of DNA repair factors and genotoxic agents. To this end, we combined the IC20 of inhibitors of eight DNA repair factors (PF‐47736 (Chk1i), NU‐7441 (DNA‐PKi), AZD‐1775 (Wee1i), AZD‐6738 (ATRi), MK‐8776 (Chk1/2i), MP‐470 (Rad51i), KU‐55933 (ATMi), PJ34 (PARP1/2i)) with doxorubicin, etoposide and 4‐OH‐Cyclophosphamide, using the U2932 cell line (ABC‐DLBCL) that is one of the most resistant to the different DDR inhibitors. We calculated the combination index (CI) for each DDR inhibitor with each genotoxic drug according to the Chou‐Talalay method [44]. A CI < 0.7 was considered as a potential synthetic lethal interaction, whereas 0.7 < CI < 1 was considered as a potentially synergistic combination (Supporting Information S1). Applying a standard threshold of 2 standard deviations below the IC50 of the genotoxic agent alone [49], four DDR inhibitors were found to be synthetic lethal or synergistic with genotoxic agents, namely the Chk1 inhibitor PF‐477736, the ATR inhibitor AZD‐6738, and the ATM inhibitor KU‐55933 with both 4‐OH‐Cyclophosphamide and etoposide, and the DNA‐PK inhibitor NU‐7441 with doxorubicin. We then tested these combinations using four cell lines: DB (GCB‐DLBCL), SUDHL10 (GCB‐DLBCL), U2932 (ABD‐DLBCL) and OCI‐LY3 (ABC‐DLBCL), which are among the most resistant to conventional genotoxic agents. First, we observed a significant decrease in the IC50 of 4‐OH‐Cyclophosphamide when combined with AZD‐6738 (ATRi) and PF‐477736 (Chki), and of doxorubicin when combined with NU‐7441 (DNA‐PKi) (Figure 4A,B). Importantly, the CI calculation of each drug combination for each cell line confirmed a strong synergistic effect in all cases, except for the combination of Chk1i with 4‐OH‐Cyclophosphamide for the DB cell line (Figure 4C).

**FIGURE 5:**
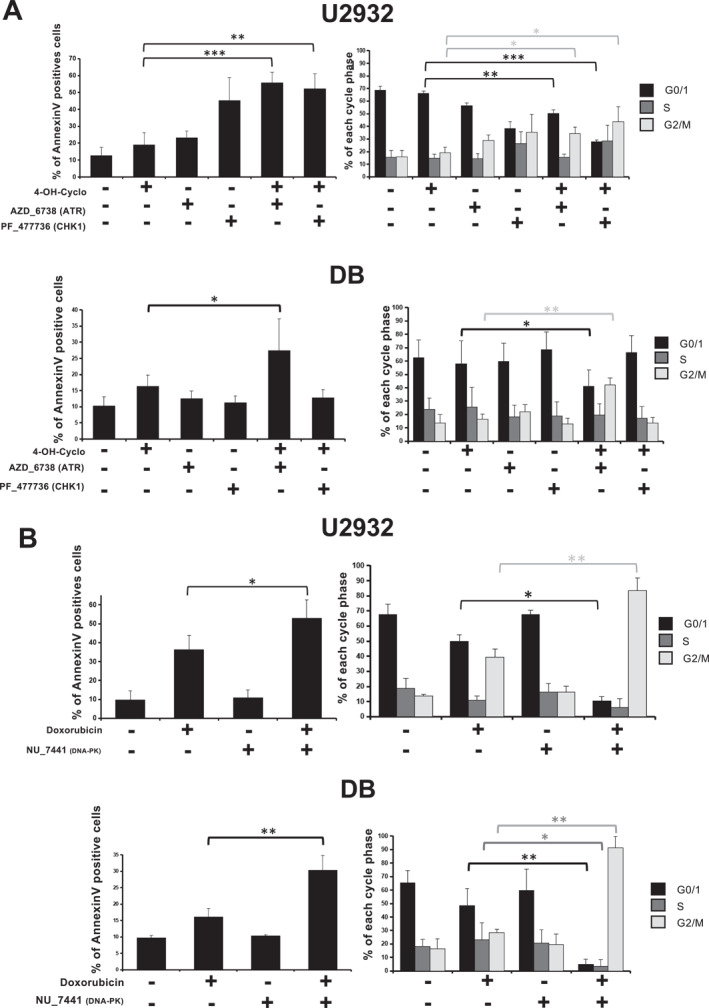
Synthetic lethal combinations enhance apoptosis, induce cell cycle defect and activate DDR. (A, B) U2932 and DB cell lines were treated as indicated with IC20 of each drug (U2932 cell line: Cyclophosphamide 1 µM, PF477736 0.31, AZD‐6738 1.01 µM, Doxorubicin 30 nM, NU7441 3.1 µM; DB cell line: Cyclophosphamide: 0.8 μM, PF477736 0.39, AZD‐6738 3.13 µM, Doxorubicin 30 nM, NU7441 3.1 µM). Apoptosis was studied by AnnexinV‐PE staining and cell cycle was studied using BrdU incorporation and DAPI after 72H of treatment. Data are the mean ± SD of three independent experiments. Statistical significance was tested using *t*‐test for pairs. *p < 0.05, **p < 0.01, ***p < 0.001, NS: non‐significant.

**FIGURE 6:**
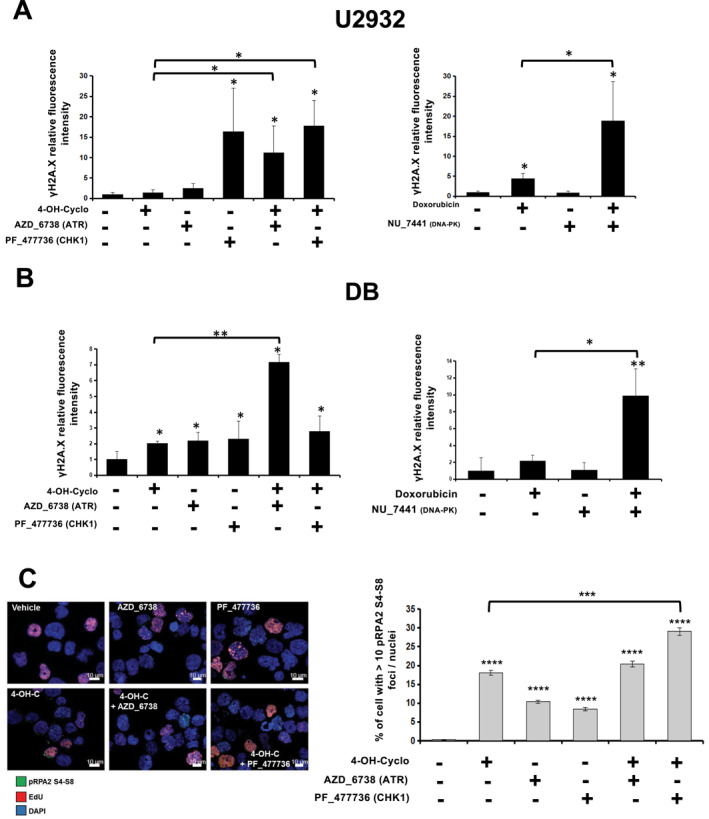
Synthetic lethal combinations induce DNA damage. (A) U2932 and (B) DB cell lines were treated as indicated on the Figure. Double strand breaks were studied by γH2A.X staining in in BrdU positive cells. Cells were analyzed by flow cytometry at 72H after treatment. Data are the mean ± SD of three independent experiments. Statistical significance was tested using *t*‐test for pairs. *p < 0.05, **p < 0.01, ***p < 0.001, NS: non‐significant. (C) U2932 cells were treated as indicated (IC50 of each drug) during 24H. At the end of the treatment, cells were fixed for immunofluorescence with 4% PFA for 10 min at RT. Foci of phospho‐RPA2 S4‐S8 were analyzed, S phase was stained by EdU and click‐it reaction and nuclei were satined with DAPI. The percentage of cells with more than 10 phospho‐RPA2 S4‐S8 foci per cell is displayed in the histogram. At least 300 cells were counted for each group. Statistical difference was tested using a Fisher's exact test. *p < 0.05, **p < 0.01, ***p < 0.001, ****p < 0.0001. Results are representative of three independent experiments. NS, non‐significant.

We further analyzed the effect of these synergistic drug combinations on cell viability using the U2932 and DB cell lines. In U2932 cells, co‐treatment with 4‐OH‐Cyclophosphamide plus ATR/Chk1 inhibitors induced a significant increase in Annexin V‐positive cells, indicative of apoptosis, and an alteration in cell cycle distribution characterized by a decrease in G0/G1 population and an accumulation in the G2/M phase (Figure 5A, upper panels). However, in the DB cell line, only the combination of 4‐OH‐Cyclophosphamide with the ATRi significantly increased the number of apoptotic cells and the inhibition of proliferation (Figure 5A, lower panels). Similar phenotypes were observed when combining doxorubicin with DNA‐PKi in both cell lines (Figure 5B). Mechanistically, co‐treatment with 4‐OH‐Cyclophosphamide and ATRi, or doxorubicin plus DNA‐PKi, increased the level of γH2AX in both cell lines (Figure 6A,B). Similarly, the level of phosphorylation of RPA2 at Serines 4 and 8 (pRPA2 S4 S8), another marker of replication stress [42], was increased by treatment with 4‐OH‐Cyclophosphamide, ATRi or Chk1i, and was further enhanced by the combination of the genotoxic agent with Chk1i (Figure 6C). Moreover, western bot analysis were perfomed to assess the effects of the ccombinations on the epression levels of several well‐known factors controlling apoptosis, cell cycle progression and DDR (Figure S5). To further characterize the impact of the drug combination on the induction of replication stress induction, we performed DNA fiber analysis. Treatment with 4‐OH‐Cyclophosphamide, AZD‐6738 (ATRi) or PF‐477736 (Chk1i) strongly reduced replication fork speed (Figure S6). Moreover, the combination of 4‐OH‐Cyclophosphamide with either inhibitor further impaired fork progression (Figure S6). We also investigated the combination of ATRi (AZD‐6738), CHK1i (PF‐477736) and ATMi (KU‐55933) IC20 with etoposide. However, no synthetic lethality or significant synergy was identified in the two DLBCL cell lines tested (Figure S7).

Taken together, these results underscore that the combination of inhibitors targeting the DDR factors ATR, Chk1 and DNA‐PK with 4‐OH‐Cyclophosphamide or doxorubicin synergizes to induce DNA replication fork stalling, leading to increased levels of DNA damage and genetic instability, which in turn leads to cell death by apoptosis.

### 3.4 ATR, Chk1 and DNA‐PK Inhibitors Synergize With Genotoxic Agents to Induce Toxicity in Primary DLBCL Cells From Patients

To confirm the therapeutic potential of the identified synthetic lethal combinations, we tested the effect of the combination of 4‐OH‐Cyclophosphamide with AZD‐6738 (ATRi) and PF‐477736 (Chk1i), and of doxorubicin with NU‐7441 (DNA‐PKi) on cell viability in primary lymph node (LN) cells coming from 6 DLBCL patients (Supporting Information S1).

**FIGURE 7:**
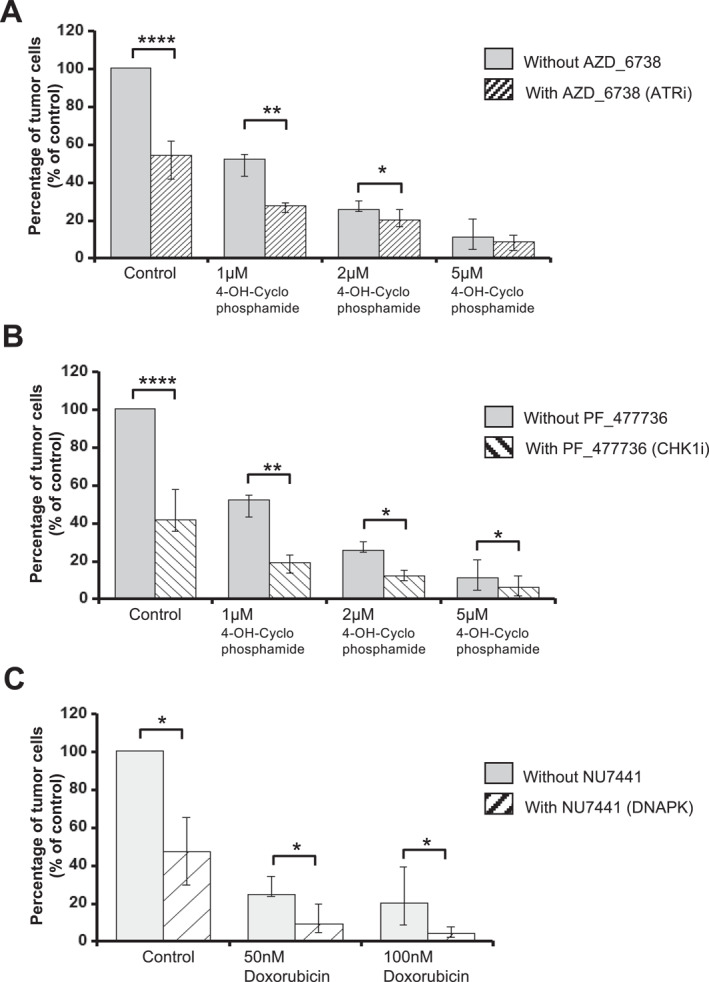
Synthetic lethal combinations have toxic effects on primary DLBCL cells. Mononuclear cells from six patients with DLBCL were cultured for 4 days in the presence of CD40 L. DLBCL cells were treated as indicated: (A) AZD‐6738, (B) PF‐477736, and (C) NU‐7441. At day 4 of culture, the viability and total cell count were assessed and the percentage of viable DLBCL cells was determined by flow cytometry. Results are median values from six different patients. Statistical significance was tested using *t*‐test for pairs. *p < 0.05, **p < 0.01, ***p < 0.001, NS: non‐.significant.

Primary DLBCL cells were co‐cultured with the non‐malignant LN microenvironment completed with 50 ng/mL of CD40 L histidine‐tagged and anti‐histidine antibodies. Primary cell cultures were treated with the drug combinations for 96 h, then counted and the fractions of viable DLBCL cells (CD19+, *κ* or *λ* +) and non‐malignant cells were determined by flow cytometry. All co‐treatments significantly reduced the number of tumor cells in cell cultures more than the genotoxic agents alone (Figure 7), confirming the synergy/synthetic lethal results obtained with DLBCL cell lines. Furthermore, the toxicity was higher in DLBCL cells compared to non‐malignant cells except for the combination of DNA‐PK/doxorubicin, which showed similar toxicity in both compartments (Figure S8). Altogether, these data suggest that the combination of ATRi or CHK1i with cyclophosphamide could be of therapeutic value in high‐risk or relpased/refractory DLBCL patients.

## 4 Discussion

Tumor heterogeneity is one of the major barriers to the development of successful cancer therapies. Activation of alternative DDR pathways to counteract therapies can lead to drug resistance. In this study, taking advantage of CRISPR‐Cas9 sreening data in DLBCL lines and focusing on genes encoding DDR factors, we identified specific dependencies on *CHEK1*, *WEE1*, *ATR*, and *RAD51* in both GCB and ABC cell line models. The combination of ATR, Chk1 and DNA‐PK inhibitors with doxorubicin or cyclophosphamide demonstrates synergistic toxicities in DLBCL cells in association with DNA damage, inhibition of proliferation and apoptosis induction.

We previously reported that high‐risk DLBCL patients are characterized by deregulation of DNA repair gene expression in association with a poor outcome after R‐CHOP treatment [16, 50]. These data support the idea that inhibitors of DNA damage signaling and DNA repair are of potential therapeutic interest in DLBCL. We found that DLBCL cells overexpressing genes involved in the FANC pathway [5] are more sensitive to ATRi, whereas overexpression of HRR or BER genes is associated with higher sensitivity to etoposide (Figure S2). More recently, it was reported that DLBCL expressing LMO2 have deficient HRR DNA repair and are more sensitive to PARP1/2 inhibitors [51]. Furthermore, PARPi synergize with chemotherapy in LMO2 positive tumor cells [51]. Here, we identified that DLBCL cell lines characterized by HRR gene overexpression [16] are more resistant to PARPi whereas NHEJ gene overexpression [16] is associated with resistance to DNA‐PK and ATM inhibitors (Figure S2). Furthermore, recent data demonstrated that CDKN2A inactivation determines sensitivity to ATRi [37]. The characterization of the CDKN2A status may be important to guide the use of ATRi in lymphoma cells. The interest of DDR pathway GEP‐based signatures in DLBCL was recently validated to develop more personalized treatments in patients who have relapsed after frontline therapies [16, 50, 52]. Furthermore, MYC deregulation generate replicative stress and DDR in DLBCL cells [53]. According to that, synthetic lethal approaches have been developed to target essential signaling pathways downstream of MYC. Chk1 and Wee1 inhibitors have been proposed as a new therapeutic approach for DLBCL with MYC deregulation [53]. Through an integrative gene expression and genetic alteration analysis, Reddy et al. revealed dysregulated DNA damage and replication genes, including Chromodomain‐Helicase‐DNA‐binding protein 1 (CHD1), ATR, and E1A‐binding protein p300 (EP300) as drivers of DLBCL [54]. Different DLBCL genetic subgroups with specific genotypic, epigenetic, and clinical characteristics were identified including a particular subgroup in EZH2 mutated patients associated with a complex deregulation of the DDR network [6].

Here, we report DLBCL‐selective synergistic killing by ATR, Chk1 and DNA‐PK inhibitors in association with conventional chemotherapy agents. Synthetic lethality strategies have shown promise in solid tumors by targeting specific genetic vulnerabilities. Two notable examples are the use of PARP inhibitors in BRCA1/2‐mutated cancers and immune checkpoint inhibitors in tumors with mismatch repair deficiency [55, 56]. Several DDR protein addictions have emerged as potential targets for cancer therapy. BRCA1 mutations increase the risk of breast and ovarian cancers [57, 58]. High expression of FANCA and FEN1 is associated with poor prognosis in pancreatic cancer [59]. PLOD1 upregulation promotes tumor growth and metastasis in lung and gastric cancers [60]. Upregulation of RAD51 indicates poor prognosis in breast, ovarian, and prostate cancers [61]. Synthetic lethality concepts are now also being harnessed in hematological malignancies like Chronic Lymphocytic Leukemia/Small Lymphocytic Lymphoma (CLL/SLL) animal models. A study demonstrated that aggressive CLL/SLL cells lacking ATM and P53 were vulnerable to PARP1 inhibition in mouse models [62]. DLBCL's complex genetic profile and aggressive nature present opportunities for exploiting synthetic lethal interactions. The disease is characterized by a high frequency of genetic alterations, including mutations in B‐cell receptor (BCR) pathway genes, MYD88, and CD79 B, sharing similarities with solid tumors where synthetic lethality therapies have shown promise. Future research could explore the application of SL approaches in DLBCL to develop personalized, targeted treatments that leverage the specific genetic vulnerabilities of this lymphoma subtype. Our research underscores the importance of assessing DDR alterations in the heterogeneous landscape of DLBCL to develop targeted synthetic lethality therapeutic strategies. However, limitations linked to toxicity should be investigated. The use of doxorubicin is limited by a well‐documented risk of cardiotoxicity, wich may manifest chronically or acutely [63]. The synergy between NU7441 and doxorubicin may be limited by the dose limitation of doxorubicin related to cardiotoxicity. Of interest, the combination of ATRi with cyclophosphamide did not demonstrated significant toxicity on normal cells in the primary samples of patients tested. Toxicity of ATR inhibitors have been investigated in murine models alone or in combination with ionizing radiotherapy [64]. Neutrophilia was observed with all ATRi tested even if ATR inhibitors did not exacerbate irradiation mediated toxicity [64]. Schmitz et al. reported that mutations in the Enhancer of Zeste Homolog 2 (EZH2) gene define a distinct subgroup with complex roles in DDR in DLBCL [6]. This subgroup demonstrates involvement in multiple aspects of DDR, including active participation in DNA repair processes and regulation of post‐DNA damage transcriptional programs [51]. More recently, we identified that iron homeostasis plays a major role in DLBCL pathophysiology [42]. Targeting iron homeostasis results in DDR, DNA double strand breaks, delayed progression of replication forks and synergistic activity with doxorubicin or targeted treatments in DLBCL cells [16]. This presents a promising avenue for developing more effective and tailored treatments for DLBCL patients.

## Author Contributions

S.O. and J.D. performed research and participated in the writing of the paper. L.A., L.D., M.A., E.G.P. and O.K.G. participated in the research. G.C. and C.H. participated in clinical data analysis and participated in the writing of the paper. C.S. participated in bioinformatic analyses. Y.L.L., A.C., O.E., P.P. and S.R. participated in the research and in the writing of the paper. J.M. and C.B. supervised the research and the writing of the paper.

## Ethics Statement

Lymph node samples were collected after patients' written informed consent in accordance with the Declaration of Helsinki and institutional research board approval from Montpellier University Hospital (HEMODIAG_2020 cohort (ID‐RCB: 2011‐A00924‐37, NCT02134574)).

## Conflicts of Interest

The authors declare no conflicts of interest.

## Peer Review

The peer review history for this article is available at https://www.webofscience.com/api/gateway/wos/peer-review/10.1002/hon.70131.

## Supporting information


Supporting Information S1

Supporting Information S2

**Figure S1:** DDR genes are essential genes in DLBCL cells.

**Figure S2:** DNA repair scores are associated with drug response in DLBCL cell lines.

**Figure S3:** Effect of genotoxic drugs on apoptosis, cell cycle and DNA damage induction.

**Figure S4:** Effect of genotoxic drugs on apoptosis, cell cycle and DNA damage protein expression.

**Figure S5:** Effect of drugs combination on apoptosis, cell cycle and DNA damage proteins expression.

**Figure S6:** Combinations of 4‐OH‐Cyclophosphamide with Chk1i and induces DNA replication stress.

**Figure S7:** Combination of DDR inhibitors with etoposide in DLBCL cells.

**Figure S8:** Mononuclear cells from six patients with DLBCL were cultured for 4 days in the presence of CD40L.

## Data Availability

The data that support the findings of this study are available on request from the corresponding author. The data are not publicly available due to privacy or ethical restrictions.
