# Supplementary material for: Synthetic Lethal Combinations of DNA Repair Inhibitors and Genotoxic Agents to Target High‐Risk Diffuse Large B Cell Lymphoma
Source: Hematol Oncol. 2025 Aug 23;43(5):e70131. doi: 10.1002/hon.70131 (PMC12374179; doi:10.1002/hon.70131)
Supplement: Supplementary file 1 — Supporting Information S1 [file HON-43-e70131-s001.pdf]

Supplementary Table S1

Identification	Inhibitory Concentration 50 (µM) at 96H											
Drug	NU7441	PJ34	Amuvatinib	KU55933	AZD_6738	AZD_1775	PF477736	AZD_8776	4-OH-C	Doxorubicine	Etoposide	Gemcitabine
Target (inhibition)	DNA-PK	PARP	Rad51	ATM	ATR	Wee1	CHK1/2	CHK1	Alkylator	Topoisomerase II		Anti-metabolite
DB	12.78	11.85	3.66	44.01	8.27	1.085	1.249	7.47	1.18	0.2570	0.675	0.008
DOHH2	10.43	16.41	2.32	35.43	1.77	0.732	0.387	5.47	0.53	0.0060	0.026	0.004
HT	4.39	13.53	10.49	31.28	3.02	0.569	0.291	9.76	3.93	0.0202	0.484	0.005
NU-DHL-1	3.16	8.09	4.53	21.18	0.43	0.089	0.410	1.13	0.31	0.0207	0.096	0.003
NUDUL-1	3.76	12.42	3.99	24.04	0.48	0.113	0.012	0.95	0.21	0.0004	0.038	0.001
OCI-LY1	4.96	10.70	11.19	28.38	0.56	0.144	0.141	1.38	0.81	0.0162	0.117	0.005
OCI-LY-19	8.15	10.68	4.22	39.93	0.28	0.083	0.250	0.39	0.08	0.0075	0.040	0.002
OCI-LY3	9.83	8.08	12.49	59.56	2.56	0.364	0.763	9.35	1.76	0.0125	0.069	0.003
OCI-LY7	4.66	15.00	8.30	25.17	0.37	0.416	0.045	1.85	3.87	0.0048	0.028	0.010
RI-1	9.99	13.98	2.99	35.01	0.90	0.158	0.013	1.97	2.97	0.0079	0.327	0.004
SU-DHL-10	7.36	24.58	10.61	20.98	1.33	0.397	0.653	4.13	13.05	0.1800	0.550	0.006
SU-DHL-4	7.20	28.59	7.27	39.55	1.09	0.388	0.260	7.99	1.44	0.0053	0.394	0.011
SU-DHL-5	4.75	17.91	6.88	38.25	0.33	0.493	0.025	0.97	0.80	0.0003	0.074	0.006
SUDHL-6	11.89	12.41	6.36	43.76	0.50	0.667	0.238	1.58	3.86	0.0093	0.065	0.006
U2932	7.52	13.90	11.69	58.19	4.21	0.394	1.375	12.77	5.35	0.0389	0.420	0.012
WSU-DLCL2	3.82	11.66	1.17	20.97	3.13	0.845	0.165	3.92	1.16	0.0129	0.150	0.006

Supplementary Table S1: Inhibitory Concentration 50 (µM) of a panel of drugs targeting DNA repair and genotoxic agents in DLBCL cell lines. Cells were seeded in 96-well plates and treated with increasing concentrations of the indicated drugs for 96 hours. Cell viability was analyzed by Cell Titer Glo (CTG) assay and IC50 was calculated for each drug and each cell line. The IC50 represented here is the mean of at least three independent experiments.

Supplementary Table S2:

Genotoxic drug/drug targeting DNA repair	PF477736 (CHK1)	NU7441 (DNA-PK)	AZD 1775 (Wee1)	AZD6738 (ATR)	MK8776 (CHK1I)	MP470 (RAD51)	KU55933 (ATM)	PJ34 (PARP1/2)
Doxorubicin	1.17	*0.57*	3.20	1.30	1.49	1.68	1.08	4.45
Etoposide	*0.72*	1.44	1.62	*0.79*	1.05	1.54	*0.83*	2.77
4-OH-cyclophosphamide	*0.70*	1.60	1.30	*0.49*	1.08	1.46	0.96	2.15

Supplementary table S2: Combination indexes of drug combinations in U2932 cell line

Supplementary Table S3:

Patient	H16-103	H16-108	H18-04	H16-223	H19-38	H18-319
Gender (M/F)	M	M	M	M	M	M
Age (Y)	66	71	78	81	88	77
Neoplasm	DLBCL	DLBCL	DLBCL	DLBCL	DLBCL	DLBCL
Type	GC (Brain)	Non-GC	Non-GC	Non-GC	Unclassified	Double-hit (MYC and BCL2)
Diag/relapse /progression	Diag	Diag	Diag	Diag	Diag	Diag
Ann Arbor stage	/	III	IV	III	IV	IV
Prognosis index	/	IPI:4	IPI:5	/	/	IPI :5
% of malignant B cells in the sample	52%	84%	23%	62%	51%	39%
% of malignant B cells in total B cells	90.6%	100%	100%	100%	100%	95%

Supplementary Table S3. Clinical data and molecular subgroup of 6 DLBCL patients used to test synthetic lethal drug combinations described in this study. M: male; F: female; Y: years.
