# Supplementary material for: Synthetic Lethal Combinations of DNA Repair Inhibitors and Genotoxic Agents to Target High‐Risk Diffuse Large B Cell Lymphoma
Source: Hematol Oncol. 2025 Aug 23;43(5):e70131. doi: 10.1002/hon.70131 (PMC12374179; doi:10.1002/hon.70131)
Supplement: Supplementary file 2 — Supporting Information S2 [file HON-43-e70131-s005.pdf]

Supplementary Figure S7: Combination of DDR inhibitors with etoposide in DLBCL cells. **(A)** DLBCL cell lines were treated with etiposide in combination with IC20 of ATR inhibitor (AZD_6738) or IC20 of CHK1 inhibitor (PF_477736) or ATM inhibitor (KU_55933). IC50 were calculated after viability assessment by CellTiter-Glo luminescent cell viability assay. Results are representative of three independent experiments. Significant synergy and combination index (CI) are calculated by the method of Chou Talalay. **(B)** Inhibitory concentration 50 of etoposide in combination with IC20 of ATR inhibitor (AZD_6738) or CHK1 inhibitor (PF_477736) or ATM inhibitor (KU_55933). IC50 were calculated after viability assessment by CellTiter-Glo luminescent cell viability assay. Results are representative of three independent experiments. **(C)** Combination index (CI) are calculated by the method of Chou Talalay. Normalized IC50 isobolograms showing the drug combination effect in the DLBCL cell lines. Statistical significance was tested using t-test for pairs. * $P<0.05$, ** $P<0.01$.
