# Supplementary material for: Synthetic Lethal Combinations of DNA Repair Inhibitors and Genotoxic Agents to Target High‐Risk Diffuse Large B Cell Lymphoma
Source: Hematol Oncol. 2025 Aug 23;43(5):e70131. doi: 10.1002/hon.70131 (PMC12374179; doi:10.1002/hon.70131)
Supplement: Supplementary file 3 — Figure S1: DDR genes are essential genes in DLBCL cells. [file HON-43-e70131-s010.pdf]

Supplementary Figure S1

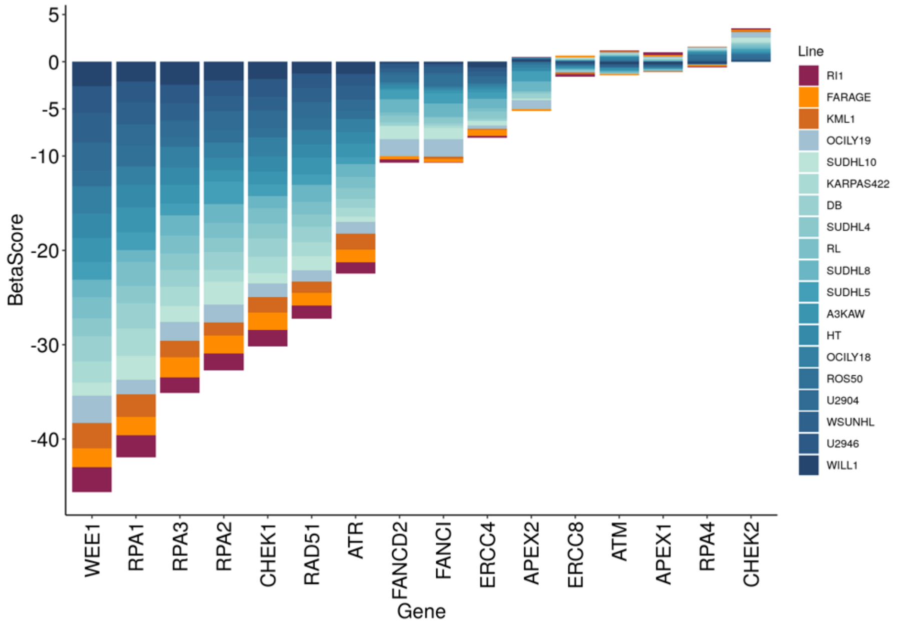

Supplementary Figure S1:

DDR genes are essential genes in DLBCL cells. Cumulative plot showing the dependency scores calculated using data from DLBCL cell lines (Cancer Depe,dency Map project; https://depmap.org). A negative score indicates that gene is more likely to essential in DLBCL cell lines.
