# Supplementary material for: Synthetic Lethal Combinations of DNA Repair Inhibitors and Genotoxic Agents to Target High‐Risk Diffuse Large B Cell Lymphoma
Source: Hematol Oncol. 2025 Aug 23;43(5):e70131. doi: 10.1002/hon.70131 (PMC12374179; doi:10.1002/hon.70131)
Supplement: Supplementary file 4 — Figure S2: DNA repair scores are associated with drug response in DLBCL cell lines. [file HON-43-e70131-s004.pdf]

# Supplementary Figure S2

A

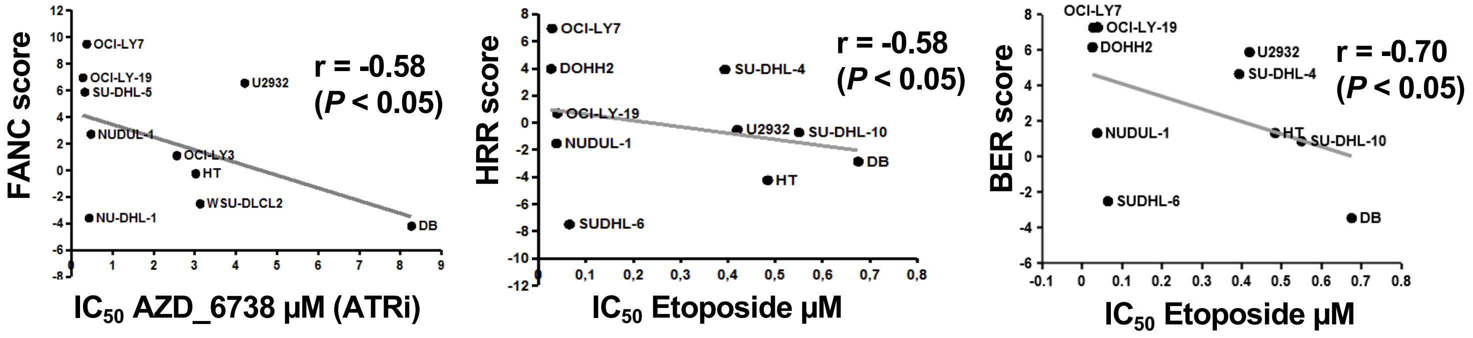


B

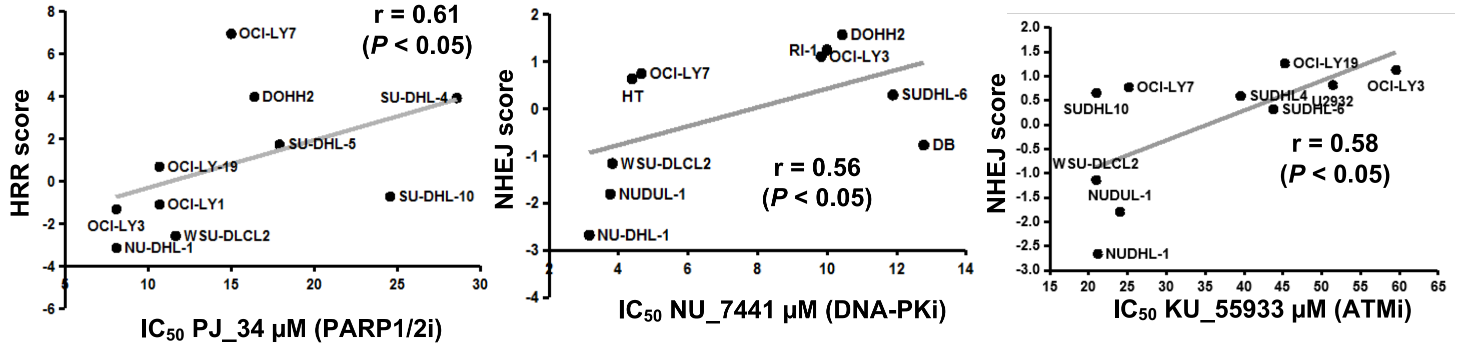


**Supplementary Figure S2: DNA repair scores are associated with drug response in DLBCL cell lines**

The graphics represent correlation between DNA repair scores (Bret C et al. BJH. 2015) and drug response with Spearman test.
