# Supplementary material for: Synthetic Lethal Combinations of DNA Repair Inhibitors and Genotoxic Agents to Target High‐Risk Diffuse Large B Cell Lymphoma
Source: Hematol Oncol. 2025 Aug 23;43(5):e70131. doi: 10.1002/hon.70131 (PMC12374179; doi:10.1002/hon.70131)
Supplement: Supplementary file 5 — Figure S3: Effect of genotoxic drugs on apoptosis, cell cycle and DNA damage induction. [file HON-43-e70131-s002.pdf]

Supplementary Figure S3:

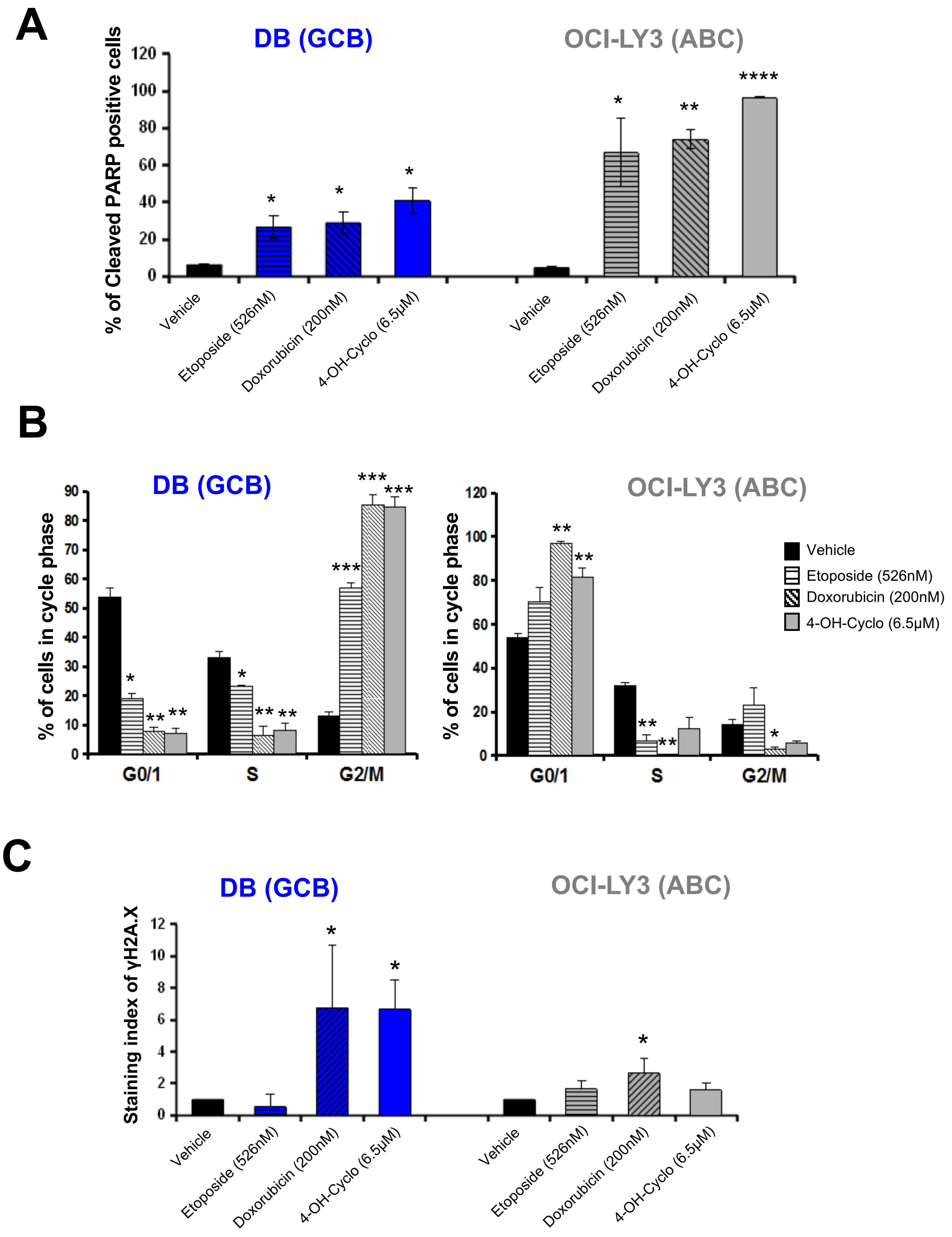

Supplementary Figure S3: Effect of genotoxic drugs on apoptosis, cell cycle and DNA damage induction. (A,B) Cells were treated as indicated for 72 hours. (A) Apoptotic cells (positive for cleaved-PARP-PE staining) were quantified by flow cytometry. **(B)** BrdU (10 μ M) was added to the culture medium during the last 1.5 hour of treatment. BrdU was detected with a specific anti-BrdU antibody and DNA was stained with DAPI. Cell cycle was analyzed based on BrdU staining and the intensity of DAPI. **(C)** DNA damage induction was anlyzed by measuring γH2AX levels in BrdU-positive cells. All data represent the mean +/- SEM of three independent experiments. Statistical significance was tested using a Student's t-test for pairs. * $P<0.05$, ** $P<0.01$.
