# Supplementary figures and images for: Synthetic Lethal Combinations of DNA Repair Inhibitors and Genotoxic Agents to Target High‐Risk Diffuse Large B Cell Lymphoma
Source: Hematol Oncol. 2025 Aug 23;43(5):e70131. doi: 10.1002/hon.70131 (PMC12374179; doi:10.1002/hon.70131)

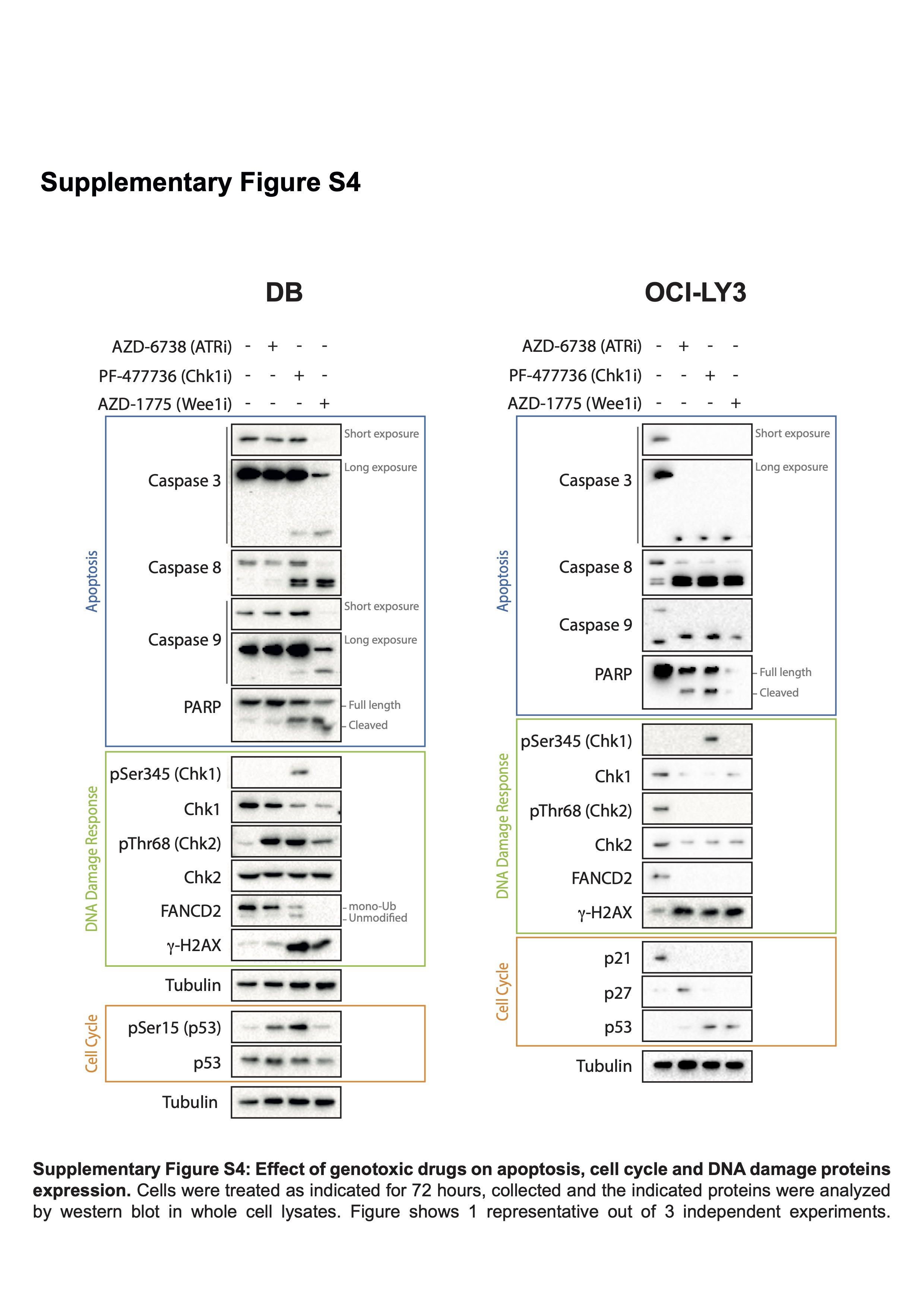

Supplement: Supplementary file 6 — Figure S4: Effect of genotoxic drugs on apoptosis, cell cycle and DNA damage protein expression. [file HON-43-e70131-s003.jpg]

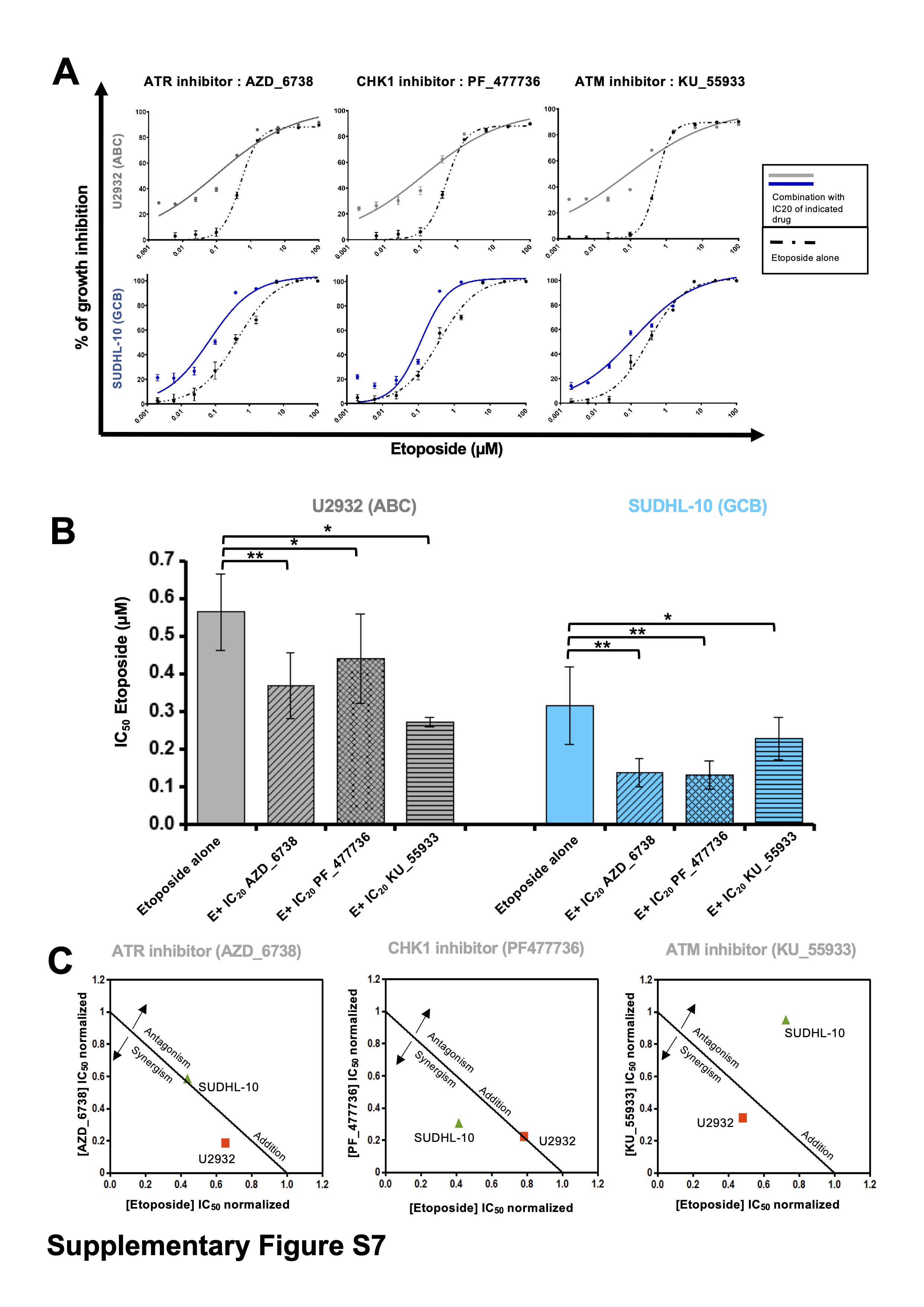

Supplement: Supplementary file 9 — Figure S7: Combination of DDR inhibitors with etoposide in DLBCL cells. [file HON-43-e70131-s009.jpg]
