# Supplementary material for: Synthetic Lethal Combinations of DNA Repair Inhibitors and Genotoxic Agents to Target High‐Risk Diffuse Large B Cell Lymphoma
Source: Hematol Oncol. 2025 Aug 23;43(5):e70131. doi: 10.1002/hon.70131 (PMC12374179; doi:10.1002/hon.70131)
Supplement: Supplementary file 7 — Figure S5: Effect of drugs combination on apoptosis, cell cycle and DNA damage proteins expression. [file HON-43-e70131-s006.pdf]

Supplementary Figure S5

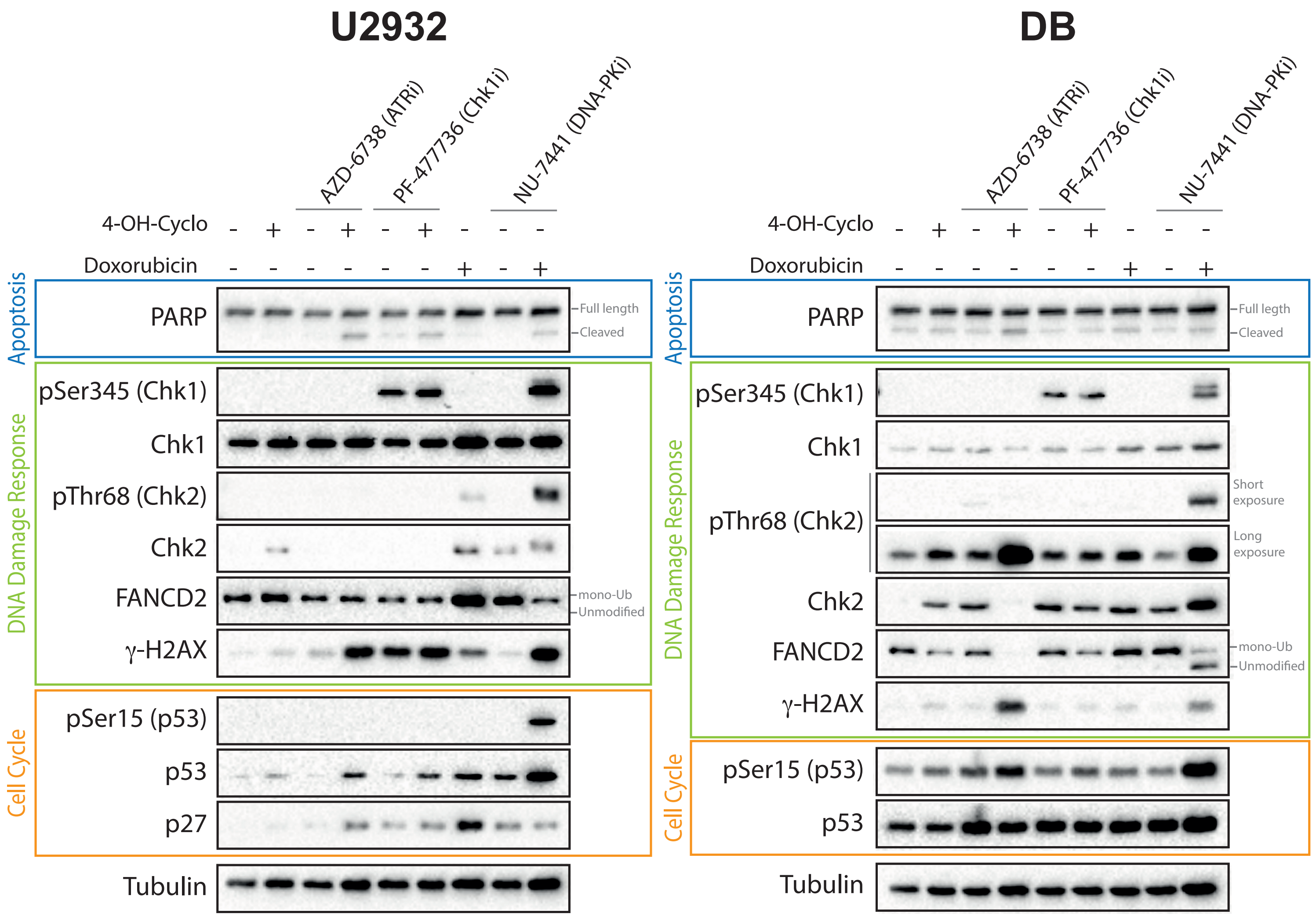

Supplementary Figure S5: Effect of drugs combination on apoptosis, cell cycle and DNA damage proteins expression. Cells were treated as indicated for 72 hours, collected and the indicated proteins were analyzed by western blot in whole cell lysates. Figure shows 1 representative out of 3 independent experiments
