# Supplementary material for: Synthetic Lethal Combinations of DNA Repair Inhibitors and Genotoxic Agents to Target High‐Risk Diffuse Large B Cell Lymphoma
Source: Hematol Oncol. 2025 Aug 23;43(5):e70131. doi: 10.1002/hon.70131 (PMC12374179; doi:10.1002/hon.70131)
Supplement: Supplementary file 8 — Figure S6: Combinations of 4‐OH‐Cyclophosphamide with Chk1i and induces DNA replication stress. [file HON-43-e70131-s007.pdf]

Supplementary Figure S6:

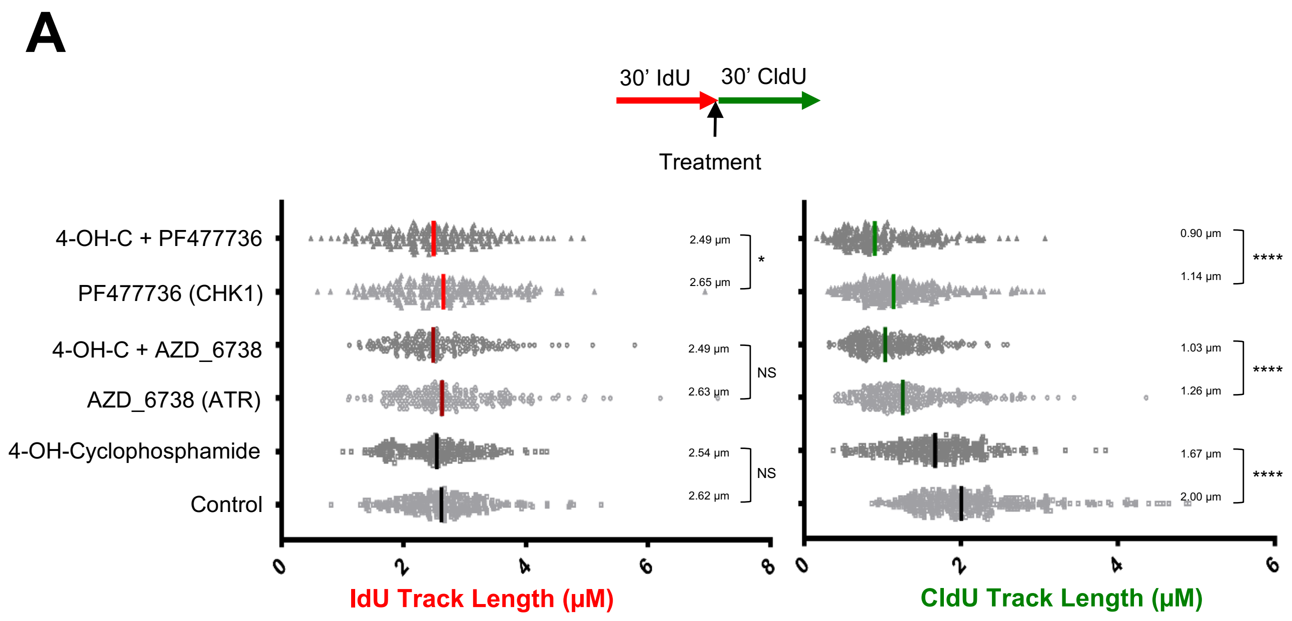

Supplementary Figure S6: Combinations of 4-OH-Cyclophosphamide with Chk1i and induces DNA replication stress. (A) U2932 cells were labeled with IdU, treated as indicated and labeled with CldU (30 minutes each), and then harvested for DNA fiber assay. Dotplot represent median of track length expressed in µm. Results represent at least 200 track measurements.* *P* < 0.05, **** *P* < 0.0001, NS: non-significant, Mann-Whitney test.
