# Supplementary material for: Synthetic Lethal Combinations of DNA Repair Inhibitors and Genotoxic Agents to Target High‐Risk Diffuse Large B Cell Lymphoma
Source: Hematol Oncol. 2025 Aug 23;43(5):e70131. doi: 10.1002/hon.70131 (PMC12374179; doi:10.1002/hon.70131)
Supplement: Supplementary file 10 — Figure S8: Mononuclear cells from six patients with DLBCL were cultured for 4 days in the presence of CD40L. [file HON-43-e70131-s008.pdf]

Supplementary Figure S8

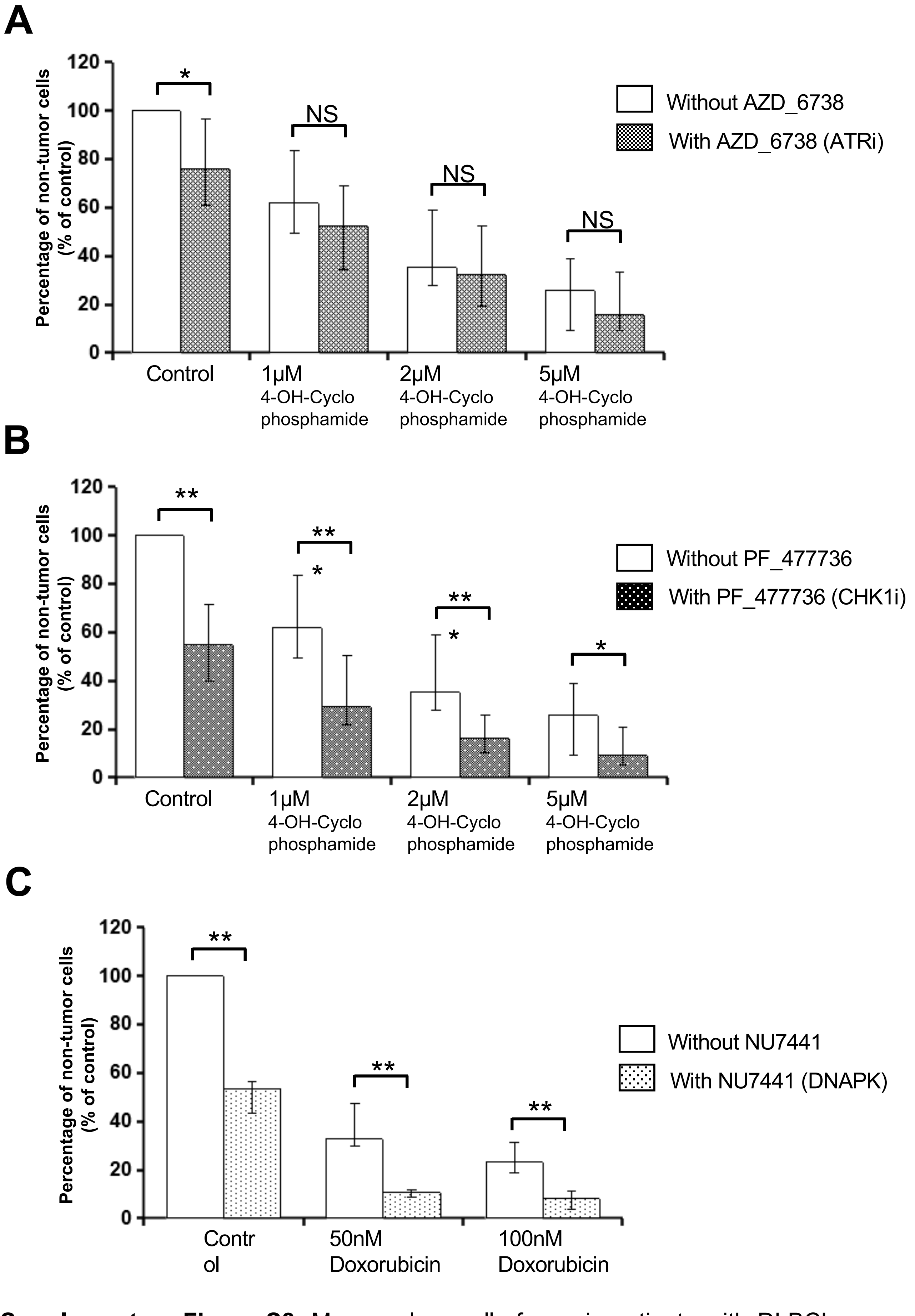

Supplementary Figure S8. Mononuclear cells from six patients with DLBCL were cultured for 4 days in the presence of CD40L. Cells were treated as indicated: **(A)** AZD-6738, **(B)** PF-477736, and **(C)** NU-7441. At day 4 of culture, the viability and total cell count were assessed and the percentage of viable non-tumor cells was determined by flow cytometry. Results are median values from six patients.
